# The Audiological Follow-Up of Children with Symptomatic Congenital Cytomegalovirus Infection: An Experience in Two Italian Centers

**DOI:** 10.3390/children10071136

**Published:** 2023-06-30

**Authors:** Silvia Palma, Francesca Forli, Cecilia Rossi, Riccardo Filice, Concetta D’adamo, Maria Federica Roversi, Daniele Monzani, Francesca Lorenzoni, Cecilia Botti, Stefano Berrettini, Luca Bruschini, Alberto Berardi, Elisabetta Genovese, Rachele Canelli

**Affiliations:** 1 Audiology, Primary Care Department, AUSL of Modena, 41100 Modena, Italy; 2 Department of Medical and Surgical Sciences for Children and Adults, Otorhinolaryngology Unit, Azienda Ospedaliero-Universitaria, 56121 Pisa, Italy; francesca.forli@unipi.it (F.F.); r.canelli@studenti.unipi.it (R.C.); 3 Department of Neonatal Intensive Care Unit, Neonatal Intensive Care Unit, Azienda Ospedaliero-Universitaria of Modena, 41125 Modena, Italy; rossi.cecilia@aou.mo.it (C.R.); riccardofilice4@gmail.com (R.F.); roversi.federica@aou.mo.it (M.F.R.); alberto.berardi@unimore.it (A.B.); 4 Department of Medical and Surgical Sciences for Children and Adults, Otorhinolaryngology Unit, Azienda Ospedaliero-Universitaria of Modena, 41125 Modena, Italy; dadamo.concetta@aou.mo.it (C.D.); elisabetta.genovese@unimore.it (E.G.); 5 ENT, Department of Surgical Sciences, Dentistry, Gynaecology and Paediatrics, University of Verona, Borgo Roma Hospital, 37100 Verona, Italy; daniele.monzani@aovr.veneto.it; 6 Division of Neonatology and NICU, Department of Clinical and Experimental Medicine, 56124 Pisa, Italy; francesca.lorenzoni@ao-pisa.toscana.it; 7 PhD Program in Clinical and Experimental Medicine, University of Modena and Reggio Emilia, 21124 Modena, Italy; cecilia.botti@unimore.it

**Keywords:** hearing loss, congenital cytomegalovirus infection, late-onset hearing loss, healthcare policy

## Abstract

Background: Congenital cytomegalovirus (cCMV) infection is the leading cause of non-hereditary sensorineural hearing loss in children. While about 10% of children reportedly display symptoms at birth, 85–90% of cCMV infection cases are asymptomatic. However, 10–15% of these asymptomatic infants may later develop hearing, visual, or neurodevelopmental impairments. This study aimed to evaluate the impact of cCMV infection on newborns’ hearing function with a particular emphasis on progressive and late-onset cases. Methods: This study is a retrospective chart analysis with longitudinal character and was conducted in two Italian centers: Center 1 (from 1 November 2007 to 31 December 2021) and Center 2 (from 1 January 2012 to 31 December 2021). Data collected included newborn hearing screening results, characterization of hearing loss (unilateral/bilateral, degree of impairment), and audiological follow-up. Results: The cohort consisted of 103 children (42% males, 58% females). In total, 28 children presented with hearing impairment; 71.4% (20 out of 28) of the cases of hearing loss were severe/profound, with 35.7% of the cases due to unilateral hearing loss. Out of twenty-eight, six experienced progression of hearing loss and four had late-onset hearing loss. Conclusions: In the absence of universal cCMV screening, hearing screening at birth for cCMV remains a critical factor for early diagnosis. A significant percentage of children affected by cCMV with normal audiological evaluations at birth is easily lost to follow-up. Close collaboration between neonatologists, pediatricians, and audiological services is fundamental to ensure timely diagnosis and treatment of cCMV-related hearing loss.

## 1. Introduction

Congenital cytomegalovirus (cCMV), regarded as the most prevalent congenital infection in humans, occurs in 0.2% to 6.1% of all live births [1,2,3]. Otologists and audiologists encounter cCMV as it remains a primary cause of non-genetic sensorineural hearing loss (SNHL) in children. The prevalence of this condition in the hearing-impaired infant population varies considerably. Although the connection between cCMV and hearing loss has been recognized for many years, the natural history of inner-ear involvement remains unclear. This is partly due to the fact that hearing impairment results from a combination of factors including genetic predisposition and perinatal and postnatal influences. Approximately 10% of children with cCMV infection present with symptoms at birth such as microcephaly, petechial rash, retinitis, thrombocytopenia, etc. [4]. Meanwhile, 85–90% of cases are asymptomatic at birth [2,5]. However, the long-term outcomes for this high percentage of clinically unaffected, yet infected, newborns, are unclear [6]. It is estimated that 10–15% of these infants may eventually develop hearing, visual, or neurodevelopmental impairments [2,5]. Nonetheless, the diagnostic criteria for symptomatic infection vary greatly in the literature and these differences may account for the varying prevalence and disease severity across different studies [7]. Considering cCMV as a whole (including both symptomatic and asymptomatic cases), up to 32% of newborns with cCMV infection may present with hearing loss [8].

Clinically, cCMV-related SNHL can be of varying severity, unilateral or bilateral, and can fluctuate, with periods of deterioration or improvement. It often manifests in early childhood [9], making the implementation of early hearing detection and surveillance programs crucial. Late diagnosis of hearing loss in children can impact language and cognitive development, depending on the severity of impairment and timing of onset [10].

Despite advancements in the field, it remains unclear which newborns are at risk of SNHL, especially in regard to late development. It was found that in newborns, the presence of petechiae at birth, periventricular cysts, or a seroconversion in the first trimester correlated with a higher risk of congenital hearing loss, though the significance of viral load as a risk factor remains unclear [3]. The results of an animal model study suggest that strial damage contributes to the initial hearing loss in congenital CMV infection [11]. Its pathogenesis is primarily observed in individuals with poor immune responses [12].

Another critical question is the necessity of extending hearing follow-up beyond six years of age, particularly in asymptomatic cases which contribute significantly to the overall disease burden associated with cCMV [6]. Delayed-onset and progression of SNHL among children with asymptomatic cCMV infection can continue into adolescence, although the risk of developing SNHL after 5 years of age seems not different than in uninfected children [13]. Therefore, an in-depth understanding of the natural history of cCMV is crucial for optimizing clinical follow-up and assessment of disease burden. Specifically, viral latency, defined as the persistence of the viral genome until it reactivates under certain conditions, appears pivotal in the development of late-onset hearing loss [14]. Nevertheless, it has been shown that CMV is susceptible to genetic variability, strongly influencing its replication and dissemination rates [14].

This study aims to evaluate the impact of cCMV infection on newborns’ hearing function, with specific attention to progression and late onset, across two centers in different Italian regions.

## 2. Materials and Methods

This study is a retrospective chart analysis with longitudinal character. Within the framework of quality improvement, we collected data concerning the audiological follow-up of children affected by cCMV infection. In order to maintain patient confidentiality, all spreadsheets submitted to the principal investigator were fully anonymized and did not include any identifiable data relating to patients or caregivers. The study periods ranged from 1 November 2007 to 31 December 2021 (center 1) and from 1 January 2012 to 31 December 2021 (center 2). These start dates correspond to the implementation of the regional newborn hearing screening (NHS) programs, which are annually monitored by the respective regional health services. Children affected by cCMV infection who were referred from other birth hospitals to the audiological centers for evaluation were also enrolled in the study. The data from their clinical evaluation at birth were analyzed separately to minimize selection bias.

### 2.1. CMV Testing

Since 2012, following the guidelines of the Italian Society of Neonatology, CMV DNA research has consistently been performed in the following cases: preterm newborns (born before the 36th week of gestational age), small-for-gestational-age babies (SGA, birth weight < 3° percentile), neonates born to CMV-infected mothers, babies showing abnormal prenatal cerebral aspects at ultrasound, babies with microcephaly (head circumference < 3° percentile), and those with persistent jaundice, thrombocytopenia, and/or neutropenia [15,16]. Beginning in 2008 in Center 1 and from 2014 in Center 2, viral DNA research was performed in all newborns that failed the newborn hearing screening. Center 1 also functions as the referral center for the entire region, receiving many children from other birth hospitals who fail the hearing screening.

Both hospital centers, as standard practice, have a professional reference for monitoring cases of cCMV infection, aiming to facilitate the organization of multidisciplinary follow-up for affected children.

Diagnosis of CMV infection was confirmed by testing urine samples using the shell vial assay (SVA). Cases of cCMV infection were defined as any newborn ≤ 14 days of age with a positive CMV urine test or with a positive polymerase chain reaction (PCR)-CMV test confirmed on Guthrie cards.

### 2.2. NHS Program

Universal newborn hearing screening allows for the early detection of hearing impairment in neonatal infants. In both regions, the NHS program is based on a two-stage protocol and is differentiated according to the Joint Committee on Infant Hearing (JCIH) guidelines, for well babies and children with risk factors for hearing [16,17].

NHS testing was performed in the Neonatal Department by audiometry technicians or nurses using a Madsen AccuScreen device (Natus^®^ Medical Incorporated, Taastrup, Denmark), which can register both otoacoustic emissions (OAEs) and Automated Auditory Brainstem Response (AABR).

Neonates without risk factors typically undergo OAE on the second day of life or before hospital discharge. The results can be classified as “pass” if there is an OAE response in both ears, and “refer” in cases of repeated unclear unilateral or bilateral response [18]. In instances of unclear responses, the neonates are referred to audiological services for a more comprehensive evaluation.

Newborns with risk factors for hearing loss such as syndromic features (e.g., atresia auris and facial dysmorphia), TORCH infections, meningitis, encephalitis, family history of hearing loss, administration of aminoglycoside or other ototoxic drugs for more than 5 days, hyperbilirubinemia treated with exchange transfusion, neonatal intensive care unit (NICU) admission for more than 5 days, birth weight < 1500 g, and gestational age < 28 weeks [19] undergo NHS through OAEs and AABR or clinical ABR.

For OAE detection, the tool utilizes a sampling frequency of 16 kHz. The stimulus is a ‘‘click’’ with an intensity ranging from 70 to 84 dB SPL (45–60 dB HL) and the intensity is automatically calibrated, depending on the volume of the ear canal. The stimulus frequency is approximately 60 Hz, and the frequency range lies between 1.5 and 4.5 kHz. For the execution of AABR, a click sequence with an intensity of 35 dB nHL is commonly employed, coupled with a pacing rate of 80 Hz and a sampling frequency of 16 kHz.

Demographic data were obtained from the Regional Health services.

### 2.3. Audiological Evaluation and Follow-Up

According to protocol, all infants with cCMV undergo evaluation at 1, 3, and 6 months, then once every 6 months until 3 years of age, and then once a year until age 6. This follow-up schedule is modified in case of a hearing-impairment diagnosis. The surveillance path provides neonatal, pediatric, ophthalmologist, and neurological controls as indicated by Italian guidelines [15].

Children undergo otoscopy, ABR, OAE, and tympanometry until they reach six months of age. The clinical ABR is recorded during spontaneous sleep, using Medelec^®^ Synergy software. The parameters evaluated include the identification of I, III, and V wave peaks at various stimulus intensities, identification of the V wave threshold expressed in dB nHL, and measurements of the peak latencies of I, III, and V waves as well as the interaural difference of V wave latency (IT-5), expressed in ms. Thresholds of V wave identification ≤ 30 dB nHL, without a pathological delay in latency, are deemed indicative of normal results.

An acoustic immittance test is conducted in order to rule out potential over-estimations of the auditory threshold caused by middle or external ear dysfunctions, which are quite common in this age group.

When children are between 8 and 12 months of age, a visual reinforcement audiometry (VRA) may be performed, depending on their compliance. This procedure is carried out through a two-channel diagnostic audiometer in Center 1 (Piano Plus VRA, Audiology and Balance, Inventis Srl, Padova, Italy) or using Astera by Madsen at Center 2.

All families with normal results are advised, as per standard practice for children at risk of hearing loss, to bring their children to the hospital for a repeat audiological evaluation at the age of one year. Long-term follow-ups are conducted in cooperation with the family pediatricians.

To better define the progression and late-onset cases, we decided to categorize [20] SNHL as congenital (diagnosed within the first month of life), early-onset (detected in the first ABR assessment from ≥1 month to 12 months of life), or delayed-onset (detected after ≥1 year assessments with normal hearing). Data collected included NHS results, characterization of hearing loss (unilateral/bilateral, degree of impairment), weeks of gestational age at birth, birth weight, gender, and any administration of antiviral therapy.

The severity of hearing loss (based on four-frequency average hearing loss in the better ear at 0.5, 1, 2, and 4 kHz) was defined according to the WHO classification: mild (≥26 to <40 dB), moderate (≥41 to <55 dB), moderate-severe (≥56 to <70 dB), severe (≥71 to <90 dB), and profound (>90 dB) [21].

## 3. Results

During the study periods, Center 1 Hospital recorded 31,620 children, of which 31,545 underwent NHS, identifying 23 cases of cCMV infection. Similarly, Center 2 Hospital recorded 29,636 children with 29,616 undergoing NHS and 30 identified with cCMV infection. The NHS coverage was over 99% in both centers [17,18].

**Figure 1:**
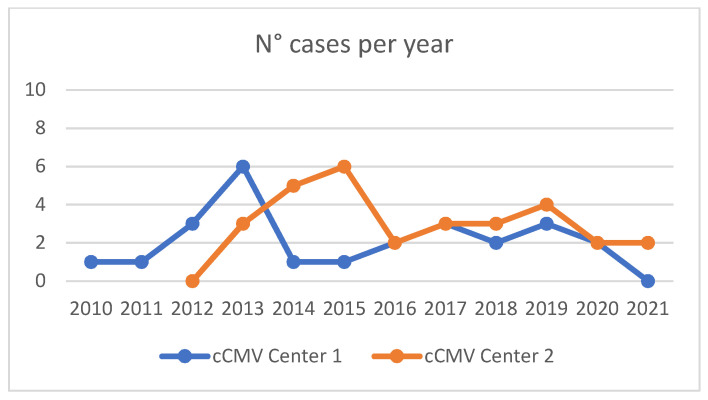
Number of cCMV cases per year in both centers. Center 1 results are in blue.

The trends of cCMV infection in newborns over time, calculated at both hospitals, are depicted in Figure 1. The prevalence of cCMV in the cohorts of children born at the two centers was calculated by dividing the number of neonates with cCMV infection by the total number of newborns. During the observation periods, the prevalence was 0.07% at Center 1 and 0.1% at Center 2. It is noteworthy that data collection at Center 2 began in 2012, a year in which not a single child was identified with cCMV. This was the same year an earthquake struck the county, leading to the operation of only three out of the five maternity wards functioning for a 4–5 month period.

Table 1 describes the clinical features of the two cohorts (n = 103 children) affected by cCMV infection. They all underwent NHS tests in the respective birth hospitals.

In Center 1, 62 children (24 males, 38 females) were enrolled (23 delivered in the center and 39 referred to the center from outside hospitals.

In Center 2, 41 children (20 males, 21 females) were enrolled (30 delivered in the center and 11 referred to the center from outside hospitals). Males represent 42% of the total population.

We classified cases as “asymptomatic” only when they did not exhibit specific alterations, including hearing loss. Also considering late-onset cases, there were 28 children with varying degrees of hearing impairment out of 103 (=27%, see also Table 2). The rate of false positive cases at OAE test was 10%. A total of 27 of 103 cases (26%) had abnormal brain findings at ultrasound or MRI.

Characteristics of hearing impairment are detailed in Table 3. The data show that 71.4% (20 out of 28) of the cases of hearing loss were severe/profound, with 35.7% of the cases due to unilateral hearing loss. In 13 out of 103 (12%) newborns, a viral infection was diagnosed as a result of them undergoing CMV testing due to NHS failure. Among these 13 cases, hearing loss was bilateral in 8 instances.

The data concerning progressive and late-onset hearing impairment cases are shown in Table 4. Out of 28, 6 children (around 21%) experienced progression of hearing loss, with 4 progressing unilaterally, and 2 bilaterally. In the cases of bilateral progression, one became of moderate-severe entity and the other became severe. Three patients affected by unilateral progression presented moderate hearing loss in the ear where progression occurred and profound hearing loss in the other ear. This led to all patients being affected by severe-deep bilateral hearing loss. Only one child presented one “normal” ear with progressively severe hearing loss in the other ear.

Overall, late-onset hearing loss occurred in 4 out of 28 children (14%).

All children who were highly suspected of hearing loss during the initial audiological examinations completed their follow-up, occasionally through other audiological services. Among the children who had normal auditory function at first examination after the NHS test (n = 79), follow-up visits were suspended within age 1 in 21 cases or age 3 years in 23 cases. In total, 43 children in this group did not complete the first three years’ follow-up. One child that presented late-onset hearing loss was lost at the first 3 years’ follow-up. Three children relocated within the first two months of life and continued their follow-up outside of the region.

## 4. Discussion

This study provides an analysis of the local clinical-epidemiological features of cCMV-related hearing loss across two different regional cohorts over a decade. Given that the literature on the demographic characteristics of children with cCMV-related hearing loss appears to be not extensive [22], it is important to gather prevalence rates from around the world. This information can enhance our understanding of the scope of the issue and aid in the organization of detection and intervention programs.

The prevalence of cases of symptomatic cCMV infection was comparable across the two centers, falling within the range reported in high-income countries, while in other experiences, studies of cCMV prevalence have identified disparities among communities [23]. This homogeneous result facilitated planning of resources dedicated to the implementation of targeted programs.

The recent neonatal guidelines recommending cCMV infection screening at birth contributed to observing shifts in the prevalence of cCMV; both centers have reached a stabilization in the annual number of cases. It is necessary to underline that in the absence of targeted or universal cCMV screening, most children are identified due to symptoms suggestive of cCMV infection [24]; in this regard, a recent position statement on the early-identification of hearing loss in cCMV infection through the NHS program has been published [25]. In recent years, the importance of newborn hearing screening in diagnosing congenital hearing loss has been demonstrated. It has also become evident that early detection of hearing loss is key to reducing the social impact and disability associated with this condition.

As indicated by the number of children affected by cCMV identified through NHS in our study, the synergy between the universal newborn hearing screening program and cCMV testing for asymptomatic infants who failed the OAE test allowed early diagnosis and the beginning of follow-up strategies [26].

Most children of the cohort had a clear hearing screening response, with different percentages between the groups born in and out of the two hospitals. This is partly due to an increase in the number of children receiving CMV testing at birth, according to neonatal guidelines, most of whom were selected as preterm. A higher percentage of unclear responses in neonates coming from other facilities could be attributed to a selection bias, as many families receiving NHS pass results likely chose to avoid unnecessary travel to other towns, especially during the pandemic. Moreover, mild hearing loss cases are not easily identified through OAE and ABR, in the presence of prematurity or low birth weight, is not always reliable at the first response. A further complication in understanding cCMV-associated hearing loss is that cCMV can only be detected with high sensitivity when neonates are tested within the first three weeks of life. It can happen that infants are identified with SNHL when they are too old for direct testing to be specific for cCMV infection. In such cases, the only means to detect cCMV infection in children with SNHL is usually through newborn dried blood spot testing.

In relation to hearing loss outcomes, our findings confirm that most early-onset cases are bilateral, as already reported [27]. A significantly high percentage is due to severe/profound hearing loss, probably attributable to the intrinsic characteristics of CMV, which is a neurotropic virus that is able to infect a lot of different types of cells by crossing the fetal blood–brain barrier [28]. Regarding this, although the inner ear is “protected” in the bony labyrinth and the cochlea is considered part of the peripheral nervous system, communication routes between intracranial spaces and the inner ear have been put in evidence [29]. Several studies found that significantly more newborns with hearing loss at birth had intracranial anomalies on ultrasonography or MRI [30,31]. We could not report all data about this in this study, but we found very similar percentages between children with hearing loss and children with intracranial anomalies.

A significant aspect to consider is the finding that the incidence of hearing loss increases with premature birth [32]. Acquired hearing loss in very preterm babies has been reported as a complication [33]. This suggests that hearing loss is a complex phenomenon and appears to be of multifactorial origin. It is also known that bilateral SNHL often has a genetic cause, which can sometimes be difficult to determine when parents, for various reasons, are unable to provide precise historical data.

SNHL can occur in 15% to 25% of all children with cCMV over the course of childhood [8]. The percentage of children that developed hearing loss was in this range, even though in this study, cases with hearing loss alone were considered symptomatic. The definition of symptomatic infection remains under debate. There are no definite diagnostic criteria to precisely identify the two conditions [34,35]. Consensus recommendations defining asymptomatic cCMV infection with isolated SNHL (with no apparent abnormalities to suggest cCMV disease, but sensorineural hearing loss (≥21 decibels) have been published [36]. Infants with isolated SNHL are generally considered to have an asymptomatic infection, although it has been argued that SNHL is a marker of central nervous system involvement and should be treated similarly to symptomatic disease [36]. Considering children with no other apparent lesions but hearing loss as symptomatic serves a functional purpose for several reasons: all cCMV infection cases must be followed independent of hearing thresholds, as hearing loss is the most frequent sequelae of cCMV infection and antiviral treatment can be initiated immediately by neonatologists after audiological evaluation. In this regard, a recent review reports that there is evidence in the literature to support antiviral treatment of children with symptomatic cCMV and hearing loss, but there is not the same evidence in relation to the potentially beneficial role of this on hearing outcome in children with isolate hearing loss [37]. A multidisciplinary approach to the treatment of all cCMV infection cases seems to be essential given the complexity of the health problems we are facing.

Currently, as there is no virological marker to predict who will develop sequelae [6], a better understanding of the natural history of the virus would be determinant in order to identify children at higher risk of developing hearing loss and to program tailored follow-up [38] through specific indicators [24]. Research has shown that the virus evolves on much shorter timescales and within individual infected subjects [39]. Moreover, CMV can establish a lifelong infection in the host [23] and a latency that can be reversed even after many years [40,41,42], with reactivation occurring under conditions of stress. There is also evidence of long-term adverse outcomes related to the induction of a chronic inflammatory cell-mediated immune response [43].

In our experience, a small but not negligible percentage of children have developed unilateral or moderate bilateral hearing loss lately. In contrast, a recent study reported 2% of case-patients developed SNHL severe enough for them to be candidates for cochlear implantation [13]. In this regard, more research is needed to understand if the antigenic variability of the virus can influence the grade of hearing loss and to predict which children will develop hearing loss and if the loss will be progressive. Nonetheless, neuroimaging abnormalities were not found to be predictive for the development of delayed-onset hearing loss [31].

In both centers, there was a large number of subjects with normal hearing tests at birth that were lost at follow-up in the first three years of life. One child who presented late-onset hearing loss was among them, which could be due to the lack of perception of danger, especially in those cases in which children did not present other symptoms. Long-term follow-up of children with cCMV is essential to identify those with late-onset hearing loss [44]. Moreover, as a recent study reported that vestibular dysfunction was more common than SNHL at 6 years of age, the inclusion of vestibular tests in the follow-up protocol for cCMV could also be considered in children with normal hearing [45].

The pandemic did not allow the implementation of surveillance programs for well- known reasons, primarily fear and limitations in accessing the health services. However, more effort should be made to improve awareness of cCMV infection risks among the population [46], as long-term follow-up of children with cCMV is essential to identify those with late-onset hearing loss. Despite the evident benefits of the NHS programs, the provision of post-neonatal pathways remains essential for the identification of hearing loss in very young children.

This study has several limitations: the high rate of losses to audiological follow-up, the influence of the pandemic, the referral bias from outside the centers, and the relatively small number of cases. Moreover, due to the lack of universal screening in Italy, most asymptomatic cCMV infections with pass results at NHS remain undiagnosed.

The strengths of this study include participation across multiple centers and the synergy between the NHS program and cCMV testing for asymptomatic infants who failed the OAE test. Another strength is the high coverage of NHS programs.

## 5. Conclusions

Testing all newborns who do not pass hearing screening at birth for CMV, in the absence of universal screening for CMV, currently remains a key factor for early diagnosis of those cases that do not present other CMV-related symptoms. Hearing loss should be considered a symptom of cCMV infection even when isolated. In our experience, late-onset hearing loss cases were more likely to be unilateral and hearing loss appeared moderate when bilateral.

A high percentage of children affected by cCMV with normal audiological evaluations at birth are easily lost at follow-up. Close collaboration between neonatologists, pediatricians, and audiological services is crucial to ensure timely diagnosis and treatment of cCMV-related hearing loss.

## Figures and Tables

**Table 1:** Clinical features of children with cCMV infection.

Clinical Features	Delivered in Center 1 Hospital N = 23	Delivered Outside Center 1 n = 39	Delivered in Center 2 Hospital N = 30	Delivered Outside Center 2 n = 11	Total n = 103
Gestational age at birth					
Premature (<36th week)	12 (52%)	11 (28%)	20 (66%)	3 (27%)	46 (45%)
Birth weight					
SGA (<3° percentile)	3	-	3	2	8 (8%)
Hearing Screening					
Pass bilaterally	17 (74%)	19 (48.7%)	25 (83%)	6 (54%)	67 (65%)
Refer bilaterally	2 (8.7%)	16 (41%)	4 (13%)	4 (36%)	26 (25%)
Refer unilateral	4 (17.3%)	4 (10%)	1 (3%)	1 (3%)	10 (10%)
Familiarity for hearing loss/syndromes	-	3(8%)		2 (18%)	5 (5%)
Symptomatic *	6 (31%)	21 (53.8%)	14 (46%)	4(36%)	45 (43%)
Antiviral Therapy	2 (8.6%)	10 (25.6%)	13 (43.3%)	4 (36.3%)	29 (28%)

Small for gestational age (SGA). * Infants with an isolated hearing loss confirmed at birth were considered symptomatic.

**Table 2:** Number of subjects with hearing impairment is reported also in relation to birthplace.

Tot 28 (27%)	Outside Center 2 n = 4/11 (36%)	Center 2 Hospital n = 7/30 (23.3%)	Outside Center 1 n = 13/39 (33.3%)	Center 1 Hospital n = 4/23 (17.3%)	N. Subjects with Hearing Impairment

**Table 3:** Hearing loss features according to entity and lateralization (unilateral/bilateral).

N. Subjects with Hearing Impairment	Center 1 n = 17	Center 2 n = 11	Tot 28/103
Bilateral Hearing Impairment	10	8	18/28 = 64.3%
Mild			
Moderate	2	3	5
Severe/Profound	8	5	13
Unilateral Hearing Impairment	7	3	10/28 = 35.7%
Mild	1		1
Moderate	1	1	2
Severe/Profound	5	2	7

**Table 4:** Hearing loss features of progressive and late-onset cases.

Hearing Loss	Subjects	Degree			Timing (Years) *			
	N°	Mild	Moderate	Severe-Deep	<1 Year	<2 Years	<3 Years	3–6 Years
Progressive	6	-						
Bilateral HL	2	-	1	1	1	1		
Unilateral HL	4	-	-	4	1	1	1	1
Late onset	4		-					
Bilateral HL	1	-	1	-	-	-	-	1
Unilateral HL	3	1		2	-	2	-	1

* Timing intervals refer to the year in which threshold stabilized or onset was observed.

## Data Availability

The data presented in this study are available from the corresponding author on request due to privacy restrictions.
